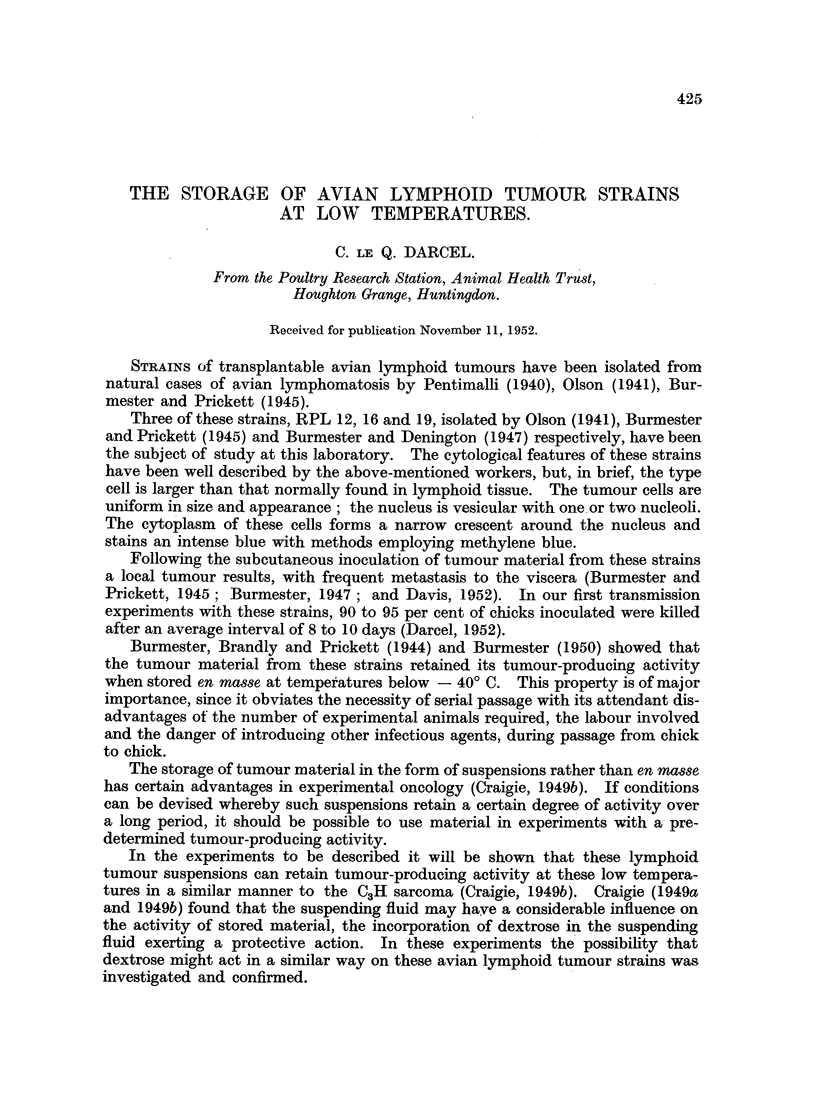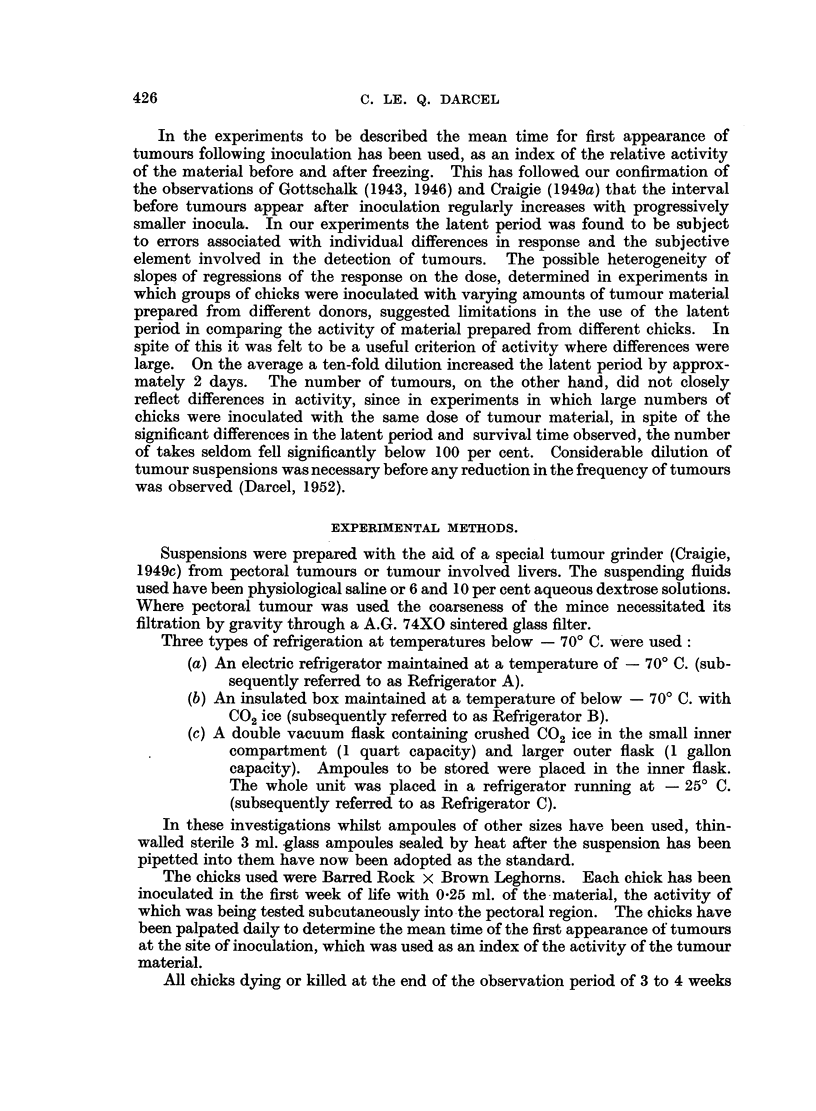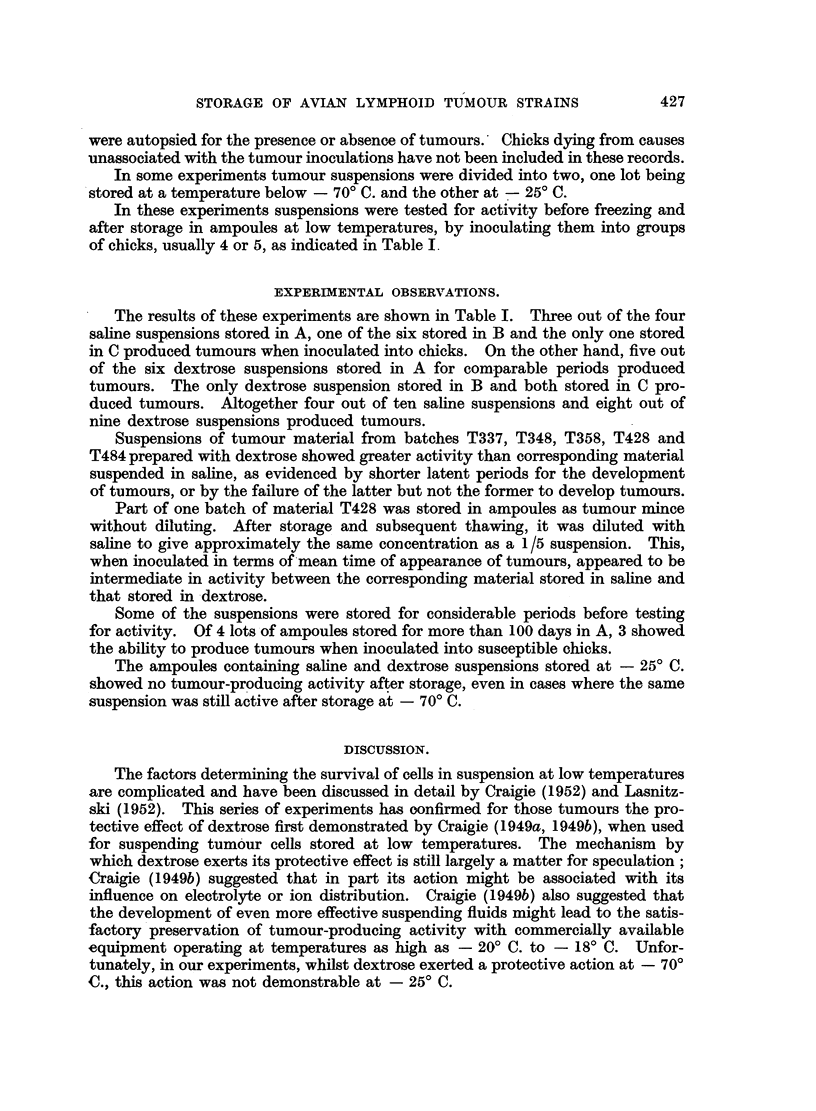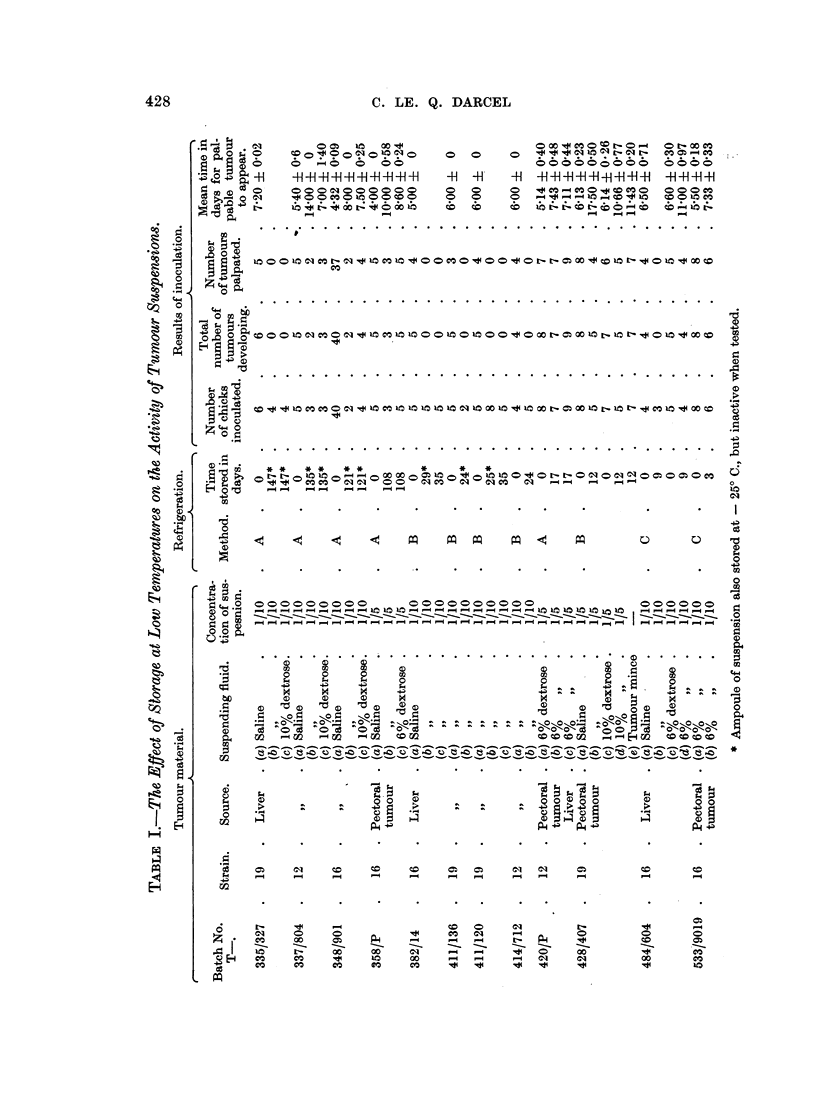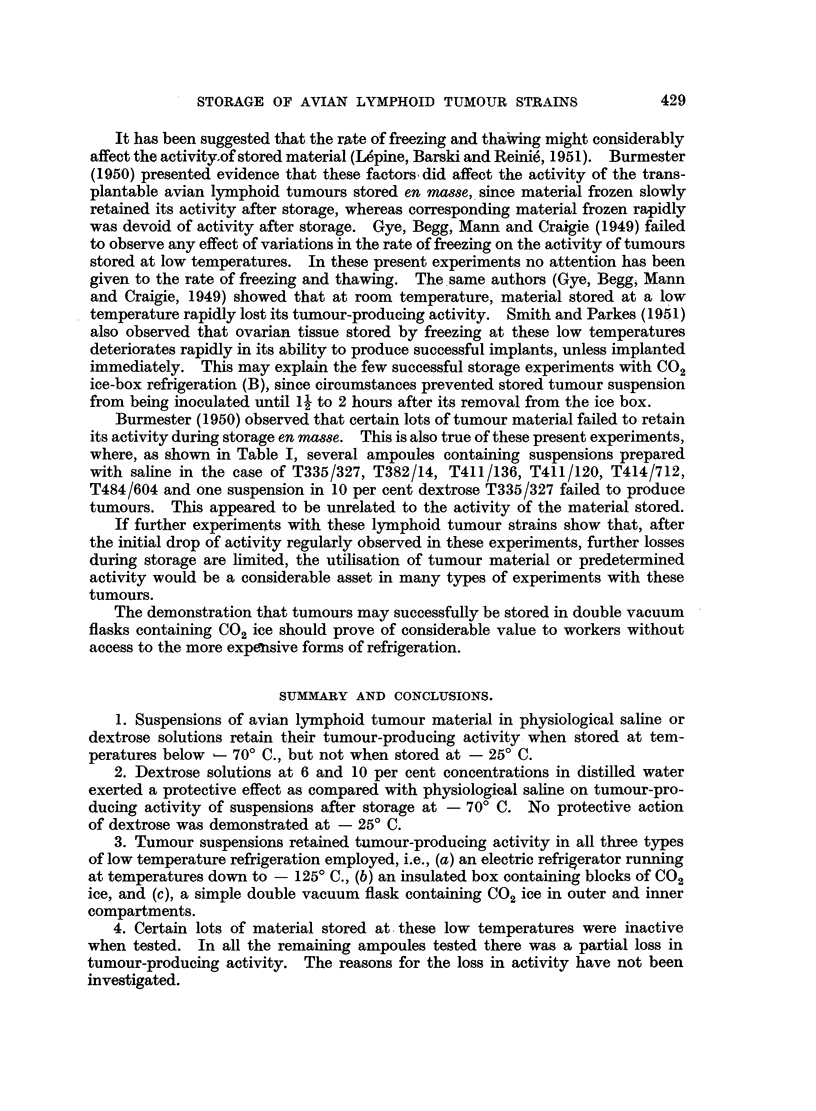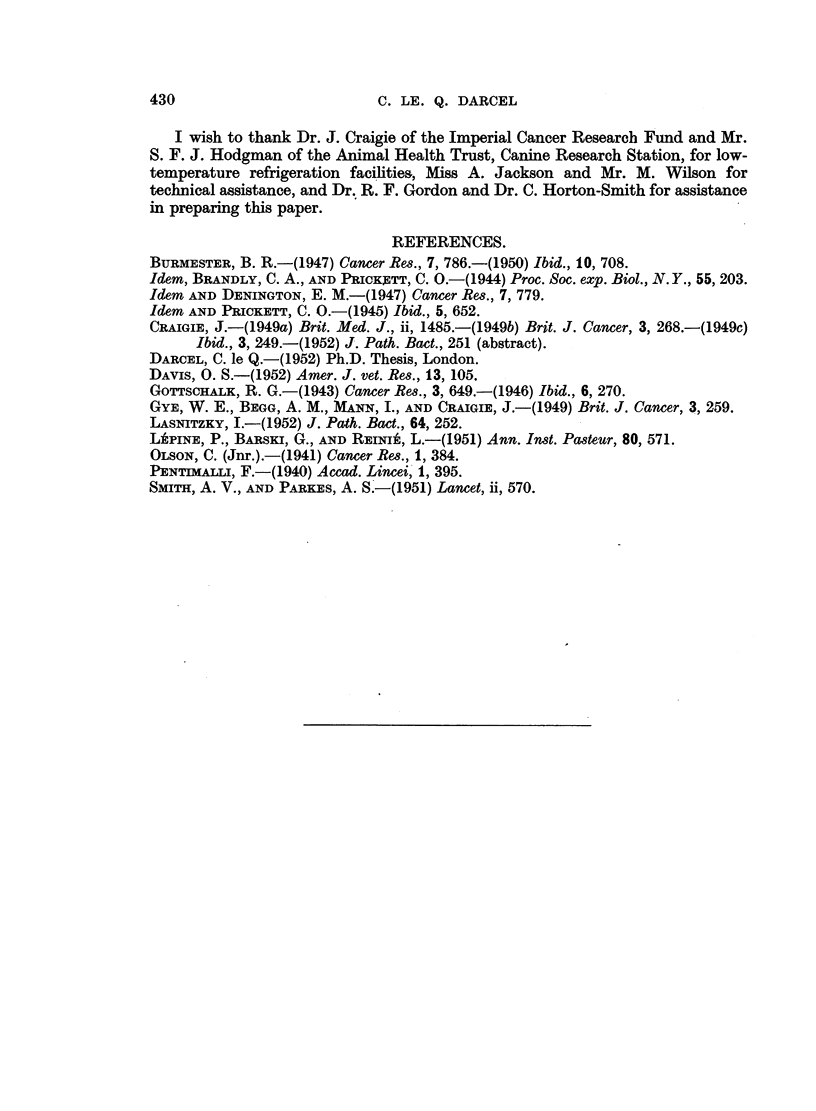# The Storage of Avian Lymphoid Tumour Strains at Low Temperatures

**DOI:** 10.1038/bjc.1952.48

**Published:** 1952-12

**Authors:** C. le Q. Darcel


### THE STORAGE OF AVIAN LYMPHOID TUMOUR STRAINS AT LOW TEMPERATURES.

C. LE Q. DARCEL.

*From the Poultry Research Station, Animal Health Trust, Houghton Grange, Huntingdon.*

Received for publication November 11, 1952.

STRAINS of transplantable avian lymphoid tumours have been isolated from natural cases of avian lymphomatosis by Pentimalli (1940), Olson (1941), Burmester and Prickett (1945).

Three of these strains, RPL 12, 16 and 19, isolated by Olson (1941), Burmester and Prickett (1945) and Burmester and Denington (1947) respectively, have been the subject of study at this laboratory. The cytological features of these strains have been well described by the above-mentioned workers, but, in brief, the type cell is larger than that normally found in lymphoid tissue. The tumour cells are uniform in size and appearance ; the nucleus is vesicular with one or two nucleoli. The cytoplasm of these cells forms a narrow crescent around the nucleus and stains an intense blue with methods employing methylene blue.

Following the subcutaneous inoculation of tumour material from these strains a local tumour results, with frequent metastasis to the viscera (Burmester and Prickett, 1945 ; Burmester, 1947 ; and Davis, 1952). In our first transmission experiments with these strains, 90 to 95 per cent of chicks inoculated were killed after an average interval of 8 to 10 days (Darcel, 1952).

Burmester, Brandly and Prickett (1944) and Burmester (1950) showed that the tumour material from these strains retained its tumour-producing activity when stored *en masse* at temperatures below $-40°$ C. This property is of major importance, since it obviates the necessity of serial passage with its attendant disadvantages of the number of experimental animals required, the labour involved and the danger of introducing other infectious agents, during passage from chick to chick.

The storage of tumour material in the form of suspensions rather than *en masse* has certain advantages in experimental oncology (Craigie, 1949*b*). If conditions can be devised whereby such suspensions retain a certain degree of activity over a long period, it should be possible to use material in experiments with a predetermined tumour-producing activity.

In the experiments to be described it will be shown that these lymphoid tumour suspensions can retain tumour-producing activity at these low temperatures in a similar manner to the $C_3H$ sarcoma (Craigie, 1949*b*). Craigie (1949*a* and 1949*b*) found that the suspending fluid may have a considerable influence on the activity of stored material, the incorporation of dextrose in the suspending fluid exerting a protective action. In these experiments the possibility that dextrose might act in a similar way on these avian lymphoid tumour strains was investigated and confirmed.

In the experiments to be described the mean time for first appearance of tumours following inoculation has been used, as an index of the relative activity of the material before and after freezing. This has followed our confirmation of the observations of Gottschalk (1943, 1946) and Craigie (1949*a*) that the interval before tumours appear after inoculation regularly increases with progressively smaller inocula. In our experiments the latent period was found to be subject to errors associated with individual differences in response and the subjective element involved in the detection of tumours. The possible heterogeneity of slopes of regressions of the response on the dose, determined in experiments in which groups of chicks were inoculated with varying amounts of tumour material prepared from different donors, suggested limitations in the use of the latent period in comparing the activity of material prepared from different chicks. In spite of this it was felt to be a useful criterion of activity where differences were large. On the average a ten-fold dilution increased the latent period by approxmately 2 days. The number of tumours, on the other hand, did not closely reflect differences in activity, since in experiments in which large numbers of chicks were inoculated with the same dose of tumour material, in spite of the significant differences in the latent period and survival time observed, the number of takes seldom fell significantly below 100 per cent. Considerable dilution of tumour suspensions was necessary before any reduction in the frequency of tumours was observed (Darcel, 1952).

### EXPERIMENTAL METHODS.

Suspensions were prepared with the aid of a special tumour grinder (Craigie, 1949*c*) from pectoral tumours or tumour involved livers. The suspending fluids used have been physiological saline or 6 and 10 per cent aqueous dextrose solutions. Where pectoral tumour was used the coarseness of the mince necessitated its filtration by gravity through a A.G. 74XO sintered glass filter.

Three types of refrigeration at temperatures below — 70° C. were used :

(*a*) An electric refrigerator maintained at a temperature of — 70° C. (subsequently referred to as Refrigerator A).

(*b*) An insulated box maintained at a temperature of below — 70° C. with $CO_2$ ice (subsequently referred to as Refrigerator B).

(*c*) A double vacuum flask containing crushed $CO_2$ ice in the small inner compartment (1 quart capacity) and larger outer flask (1 gallon capacity). Ampoules to be stored were placed in the inner flask. The whole unit was placed in a refrigerator running at — 25° C. (subsequently referred to as Refrigerator C).

In these investigations whilst ampoules of other sizes have been used, thin-walled sterile 3 ml. glass ampoules sealed by heat after the suspension has been pipetted into them have now been adopted as the standard.

The chicks used were Barred Rock × Brown Leghorns. Each chick has been inoculated in the first week of life with 0·25 ml. of the material, the activity of which was being tested subcutaneously into the pectoral region. The chicks have been palpated daily to determine the mean time of the first appearance of tumours at the site of inoculation, which was used as an index of the activity of the tumour material.

All chicks dying or killed at the end of the observation period of 3 to 4 weeks were autopsied for the presence or absence of tumours. Chicks dying from causes unassociated with the tumour inoculations have not been included in these records.

In some experiments tumour suspensions were divided into two, one lot being stored at a temperature below — 70° C. and the other at — 25° C.

In these experiments suspensions were tested for activity before freezing and after storage in ampoules at low temperatures, by inoculating them into groups of chicks, usually 4 or 5, as indicated in Table I.

### EXPERIMENTAL OBSERVATIONS.

The results of these experiments are shown in Table I. Three out of the four saline suspensions stored in A, one of the six stored in B and the only one stored in C produced tumours when inoculated into chicks. On the other hand, five out of the six dextrose suspensions stored in A for comparable periods produced tumours. The only dextrose suspension stored in B and both stored in C produced tumours. Altogether four out of ten saline suspensions and eight out of nine dextrose suspensions produced tumours.

Suspensions of tumour material from batches T337, T348, T358, T428 and T484 prepared with dextrose showed greater activity than corresponding material suspended in saline, as evidenced by shorter latent periods for the development of tumours, or by the failure of the latter but not the former to develop tumours.

Part of one batch of material T428 was stored in ampoules as tumour mince without diluting. After storage and subsequent thawing, it was diluted with saline to give approximately the same concentration as a 1/5 suspension. This, when inoculated in terms of mean time of appearance of tumours, appeared to be intermediate in activity between the corresponding material stored in saline and that stored in dextrose.

Some of the suspensions were stored for considerable periods before testing for activity. Of 4 lots of ampoules stored for more than 100 days in A, 3 showed the ability to produce tumours when inoculated into susceptible chicks.

The ampoules containing saline and dextrose suspensions stored at — 25° C. showed no tumour-producing activity after storage, even in cases where the same suspension was still active after storage at — 70° C.

### DISCUSSION.

The factors determining the survival of cells in suspension at low temperatures are complicated and have been discussed in detail by Craigie (1952) and Lasnitzski (1952). This series of experiments has confirmed for those tumours the protective effect of dextrose first demonstrated by Craigie (1949*a*, 1949*b*), when used for suspending tumour cells stored at low temperatures. The mechanism by which dextrose exerts its protective effect is still largely a matter for speculation ; Craigie (1949*b*) suggested that in part its action might be associated with its influence on electrolyte or ion distribution. Craigie (1949*b*) also suggested that the development of even more effective suspending fluids might lead to the satisfactory preservation of tumour-producing activity with commercially available equipment operating at temperatures as high as — 20° C. to — 18° C. Unfortunately, in our experiments, whilst dextrose exerted a protective action at — 70° C., this action was not demonstrable at — 25° C.

TABLE I.—*The Effect of Storage at Low Temperatures on the Activity of Tumour Suspensions.*

| Tumour material. | | | | | Refrigeration. | | Results of inoculation. | | | |
|---|---|---|---|---|---|---|---|---|---|---|
| Batch No. T—. | Strain. | Source. | Suspending fluid. | Concentration of suspesnion. | Method. | Time stored in days. | Number of chicks inoculated. | Total number of tumours developing. | Number of tumours palpated. | Mean time in days for palpable tumour to appear. |
| 335/327 | 19 | Liver | (*a*) Saline | 1/10 | A | 0 | 6 | 6 | 5 | 7·20 ± 0·02 |
| | | | (*b*) ,, | 1/10 | | 147* | 4 | 0 | 0 | |
| | | | (*c*) 10% dextrose | 1/10 | | 147* | 4 | 0 | 0 | |
| 337/804 | 12 | ,, | (*a*) Saline | 1/10 | A | 0 | 5 | 5 | 5 | 5·40 ± 0·6 |
| | | | (*b*) ,, | 1/10 | | 135* | 3 | 2 | 2 | 14·00 ± 0 |
| | | | (*c*) 10% dextrose | 1/10 | | 135* | 3 | 3 | 3 | 7·00 ± 1·40 |
| 348/901 | 16 | ,, | (*a*) Saline | 1/10 | A | 0 | 40 | 40 | 37 | 4·32 ± 0·09 |
| | | | (*b*) ,, | 1/10 | | 121* | 2 | 2 | 2 | 8·00 ± 0 |
| | | | (*c*) 10% dextrose | 1/10 | | 121* | 4 | 4 | 4 | 7.50 ± 0·25 |
| 358/P | 16 | Pectoral tumour | (*a*) Saline | 1/5 | A | 0 | 5 | 5 | 5 | 4·00 ± 0 |
| | | | (*b*) ,, | 1/5 | | 108 | 3 | 3 | 3 | 10·00 ± 0·58 |
| | | | (*c*) 6% dextrose | 1/5 | | 108 | 5 | 5 | 5 | 8·60 ± 0·24 |
| 382/14 | 16 | Liver | (*a*) Saline | 1/10 | B | 0 | 5 | 5 | 4 | 5·00 ± 0 |
| | | | (*b*) ,, | 1/10 | | 29* | 5 | 0 | 0 | |
| | | | (*c*) ,, | 1/10 | | 35 | 5 | 0 | 0 | |
| 411/136 | 19 | ,, | (*a*) ,, | 1/10 | B | 0 | 5 | 5 | 3 | 6·00 ± 0 |
| | | | (*b*) ,, | 1/10 | | 24* | 2 | 0 | 0 | |
| 411/120 | 19 | ,, | (*a*) ,, | 1/10 | B | 0 | 5 | 5 | 4 | 6·00 ± 0 |
| | | | (*b*) ,, | 1/10 | | 25* | 8 | 0 | 0 | |
| | | | (*c*) ,, | 1/10 | | 35 | 5 | 0 | 0 | |
| 414/712 | 12 | ,, | (*a*) ,, | 1/10 | B | 0 | 4 | 4 | 4 | 6·00 ± 0 |
| | | | (*b*) ,, | 1/10 | | 24 | 5 | 0 | 0 | |
| 420/P | 12 | Pectoral tumour | (*a*) 6% dextrose | 1/5 | A | 0 | 8 | 8 | 7 | 5·14 ± 0·40 |
| | | | (*b*) 6% ,, | 1/5 | | 17 | 7 | 7 | 7 | 7·43 ± 0·48 |
| | | Liver | (*c*) 6% ,, | 1/5 | | 17 | 9 | 9 | 9 | 7·11 ± 0·44 |
| 428/407 | 19 | Pectoral tumour | (*a*) Saline | 1/5 | B | 0 | 8 | 8 | 8 | 6·13 ± 0·23 |
| | | | (*b*) ,, | 1/5 | | 12 | 5 | 5 | 4 | 17·50 ± 0·50 |
| | | | (*c*) 10% dextrose | 1/5 | | 0 | 7 | 7 | 6 | 6·14 ± 0·26 |
| | | | (*d*) 10% ,, | 1/5 | | 12 | 5 | 5 | 5 | 10·66 ± 0·77 |
| | | | (*e*) Tumour mince | — | | 12 | 7 | 7 | 7 | 11·43 ± 0·20 |
| 484/604 | 16 | Liver | (*a*) Saline | 1/10 | C | 0 | 4 | 4 | 4 | 6·50 ± 0·71 |
| | | | (*b*) ,, | 1/10 | | 9 | 3 | 0 | 0 | |
| | | | (*c*) 6% dextrose | 1/10 | | 0 | 5 | 5 | 5 | 6·60 ± 0·30 |
| | | | (*d*) 6% ,, | 1/10 | | 9 | 4 | 4 | 4 | 11·00 ± 0·97 |
| 533/9019 | 16 | Pectoral tumour | (*a*) 6% ,, | 1/10 | C | 0 | 8 | 8 | 8 | 5·50 ± 0·18 |
| | | | (*b*) 6% ,, | 1/10 | | 3 | 6 | 6 | 6 | 7·33 ± 0·33 |

* Ampoule of suspension also stored at — 25° C., but inactive when tested.

It has been suggested that the rate of freezing and thawing might considerably affect the activity of stored material (Lépine, Barski and Reinié, 1951). Burmester (1950) presented evidence that these factors did affect the activity of the transplantable avian lymphoid tumours stored *en masse*, since material frozen slowly retained its activity after storage, whereas corresponding material frozen rapidly was devoid of activity after storage. Gye, Begg, Mann and Craigie (1949) failed to observe any effect of variations in the rate of freezing on the activity of tumours stored at low temperatures. In these present experiments no attention has been given to the rate of freezing and thawing. The same authors (Gye, Begg, Mann and Craigie, 1949) showed that at room temperature, material stored at a low temperature rapidly lost its tumour-producing activity. Smith and Parkes (1951) also observed that ovarian tissue stored by freezing at these low temperatures deteriorates rapidly in its ability to produce successful implants, unless implanted immediately. This may explain the few successful storage experiments with $CO_2$ ice-box refrigeration (B), since circumstances prevented stored tumour suspension from being inoculated until 1½ to 2 hours after its removal from the ice box.

Burmester (1950) observed that certain lots of tumour material failed to retain its activity during storage *en masse*. This is also true of these present experiments, where, as shown in Table I, several ampoules containing suspensions prepared with saline in the case of T335/327, T382/14, T411/136, T411/120, T414/712, T484/604 and one suspension in 10 per cent dextrose T335/327 failed to produce tumours. This appeared to be unrelated to the activity of the material stored.

If further experiments with these lymphoid tumour strains show that, after the initial drop of activity regularly observed in these experiments, further losses during storage are limited, the utilisation of tumour material or predetermined activity would be a considerable asset in many types of experiments with these tumours.

The demonstration that tumours may successfully be stored in double vacuum flasks containing $CO_2$ ice should prove of considerable value to workers without access to the more expensive forms of refrigeration.

### SUMMARY AND CONCLUSIONS.

1. Suspensions of avian lymphoid tumour material in physiological saline or dextrose solutions retain their tumour-producing activity when stored at temperatures below − 70° C., but not when stored at − 25° C.

2. Dextrose solutions at 6 and 10 per cent concentrations in distilled water exerted a protective effect as compared with physiological saline on tumour-producing activity of suspensions after storage at − 70° C. No protective action of dextrose was demonstrated at − 25° C.

3. Tumour suspensions retained tumour-producing activity in all three types of low temperature refrigeration employed, i.e., (*a*) an electric refrigerator running at temperatures down to − 125° C., (*b*) an insulated box containing blocks of $CO_2$ ice, and (*c*), a simple double vacuum flask containing $CO_2$ ice in outer and inner compartments.

4. Certain lots of material stored at these low temperatures were inactive when tested. In all the remaining ampoules tested there was a partial loss in tumour-producing activity. The reasons for the loss in activity have not been investigated.

I wish to thank Dr. J. Craigie of the Imperial Cancer Research Fund and Mr. S. F. J. Hodgman of the Animal Health Trust, Canine Research Station, for low-temperature refrigeration facilities, Miss A. Jackson and Mr. M. Wilson for technical assistance, and Dr. R. F. Gordon and Dr. C. Horton-Smith for assistance in preparing this paper.

### REFERENCES.

Burmester, B. R.—(1947) *Cancer Res.*, **7**, 786.—(1950) *Ibid.*, **10**, 708.
*Idem*, Brandly, C. A., and Prickett, C. O.—(1944) *Proc. Soc. exp. Biol., N.Y.*, **55**, 203.
*Idem* and Denington, E. M.—(1947) *Cancer Res.*, **7**, 779.
*Idem* and Prickett, C. O.—(1945) *Ibid.*, **5**, 652.
Craigie, J.—(1949*a*) *Brit. Med. J.*, ii, 1485.—(1949*b*) *Brit. J. Cancer*, **3**, 268.—(1949*c*) *Ibid.*, **3**, 249.—(1952) *J. Path. Bact.*, 251 (abstract).
Darcel, C. le Q.—(1952) Ph.D. Thesis, London.
Davis, O. S.—(1952) *Amer. J. vet. Res.*, **13**, 105.
Gottschalk, R. G.—(1943) *Cancer Res.*, **3**, 649.—(1946) *Ibid.*, **6**, 270.
Gye, W. E., Begg, A. M., Mann, I., and Craigie, J.—(1949) *Brit. J. Cancer*, **3**, 259.
Lasnitzky, I.—(1952) *J. Path. Bact.*, **64**, 252.
Lépine, P., Barski, G., and Reinié, L.—(1951) *Ann. Inst. Pasteur*, **80**, 571.
Olson, C. (Jnr.).—(1941) *Cancer Res.*, **1**, 384.
Pentimalli, F.—(1940) *Accad. Lincei*, **1**, 395.
Smith, A. V., and Parkes, A. S.—(1951) *Lancet*, ii, 570.